# On the Research Front: The NIAAA Intramural Research Program

**Published:** 1995

**Authors:** Markku Linnoila, Theodore R. Colburn, Robert C. Petersen

**Affiliations:** Markku Linnoila, M.D., Ph.D., is the director of the Division of Intramural Clinical and Biological Research at NIAAA, Bethesda, Maryland. Theodore R. Colburn, Ph.D., is the deputy director of the Division of Intramural Clinical and Biological Research at NIAAA, Bethesda, Maryland. Robert C. Petersen, Ph.D., is a consultant to NIAAA and a former assistant research director at the National Institute on Drug Abuse in Rockville, Maryland

**Keywords:** AOD dependence, AOD effects (AODE), basic research, clinical study, government agency, education and training

## Abstract

Intramural research at the National Institute on Alcohol Abuse and Alcoholism delves into many aspects of biological science relating to alcoholism, from the genetics of alcoholism to the imaging of alcohol-abusing patients’ brains. Drawing on the unique resources within the National Institutes of Health, the Intramural Research Program plans to continue its commitment to conducting cutting-edge research.

Acting under a research mandate from Congress, the National Institute on Alcohol Abuse and Alcoholism (NIAAA) has established broad research goals for itself (see box, p. 61). These entail understanding the physiological and environmental causes of alcoholism, elucidating the biological mechanisms through which alcohol affects humans, and developing effective methods for treating the disease of alcoholism and for preventing alcohol abuse. NIAAA’s efforts to reach these goals are embodied in two research arms, the Intramural Research Program (IRP), which operates within NIAAA, and the Extramural Research Program (ERP), which distributes Federal research funds to universities and other non-Federal institutions. Approximately 11 percent of the total NIAAA budget is devoted to the IRP; this percentage represents the average for all Institutes within the National Institutes of Health (NIH).

**The Intramural Research Program’s Main Goals**

Train new scientists in the field of alcohol-related research.

Identify the genetic components that predispose people to developing alcohol problems.

Determine the interactions between genetic vulnerability and environmental factors conducive to or protective of individuals at risk of developing alcohol-related problems.

Understand the interaction of alcohol with the many other factors playing causative roles in the development of alcohol abuse and alcoholism at the molecular, cellular, and whole organism levels.

Describe alcohol’s acute and long-term effects on the cellular and molecular levels of the central nervous system and other body systems.

Learn the role of nutrition in alcohol’s effects on the health of the alcoholic and of the developing fetus.

Devise more accurate diagnostic techniques and more effective preventions and interventions.

The IRP and ERP research programs have similar functions but differ in several important ways. Both are dedicated to meeting NIAAA’s research goals. The ERP concentrates on supporting independent research projects nationwide and internationally, whereas the IRP, located on the NIH campus in Bethesda, MD, conducts research employing NIAAA’s own scientists. This article focuses on the IRP’s contribution to alcoholism research by reviewing the program’s current projects, its efforts to train scientists, and the directions for its future research.

## Unique Advantages of the IRP

**Figure 1:**
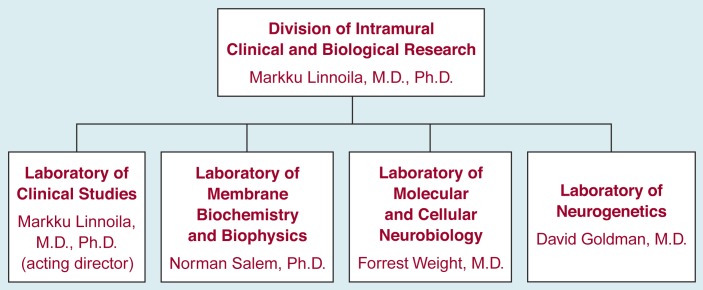
The structure of the Intramural Research Program and names of the current lab directors at the National Institute on Alcohol Abuse and Alcoholism.

The IRP conducts both basic research, aimed at understanding alcohol’s effects on cells and organs, and clinical investigations at its NIH facilities. The program comprises four laboratories, three that are concerned primarily with studying alcohol’s effects at the molecular, cellular, and organ levels and one that focuses much of its research on alcoholic patients (figure 1).

### Collaboration

Compared with the extramural community, the IRP offers certain unique opportunities. On the NIH campus, NIAAA intramural scientists interact daily with many internationally known basic and clinical researchers (there are more than 3,100 doctoral-level intramural scientists, including M.D.’s and Ph.D.’s, at NIH) and are surrounded by one of the most comprehensive concentrations of medical research talent, expertise, laboratories, and equipment in the world. The complications of alcohol abuse and alcoholism affect most of the organ systems studied in the other NIH Institutes and contribute significantly to many health problems. Accordingly, the IRP formally collaborates with intramural scientists in many of the other Institutes, including the National Cancer Institute and the National Institute of Child Health and Human Development.

Basic and clinical researchers within the IRP also collaborate with each other. Consequently, discoveries made in the laboratory can be incorporated rapidly and cost-effectively into clinical research projects, and hypotheses derived from clinical observations can be tested using basic research paradigms under the more easily controlled conditions of the laboratory.

### Training

The future of alcohol research depends on the continuing dedication of talented young researchers. Since its inception, the IRP has been committed to training. Intramural researchers serve as mentors to young scientists, providing them with access to well-equipped laboratories and the latest research technology.

The IRP provides multiple levels of training programs, ranging from projects for promising high school and undergraduate college students to programs for advanced postdoctoral fellows. Special efforts have been made to encourage talented students from families of limited means or minority origins, who often are unaware of the potential for a career in alcohol research, to join the program. Younger students often participate in summer programs or on a part-time basis during the school year under the direction of senior researchers who ensure that the students become meaningfully involved in research. In addition, medical students, as well as postdoctoral, graduate, and undergraduate students in other health specialties, receive training designed to encourage a future commitment to the alcohol research field.

#### Clinical Staff Fellowships

The Clinical Staff Fellowship Program provides training in clinical and laboratory settings for physicians early in their careers who are interested in the relationship of excessive alcohol consumption to such diverse areas as internal medicine, neurology, pediatrics, and psychiatry. In the health sciences area, efforts are made to attract promising young doctoral-level scientists both from the United States and abroad to work with NIAAA scientists on projects of shared interest. Research exchange opportunities also are available for senior scientists outside NIAAA to collaborate with IRP researchers.

#### International Training Program

The international program trains postdoctoral scientists; promotes collaborations with senior foreign scientists; and successfully facilitates the mutual exchange of ideas, skills, and cross-cultural research. Participating researchers range from promising junior scientists with less than 3 years of postdoctoral experience to senior scientists who are internationally recognized. The participation period may range from several months to several years. Currently a total of 37 fellows from 19 countries work in the intramural laboratories under the auspices of this program’s visiting arm. Intramural scientists also are engaged in 11 international collaborative projects with scientists in 8 countries. For example, in one major project located in Finland, researchers are studying alcoholism and impulsive behavior among violent offenders.

## Quality Control of Intramural Science

Unlike the ERP-sponsored projects, intramural projects are not peer reviewed in advance by outside experts but are reviewed carefully and continuously by senior NIAAA intramural investigators. In addition, the NIAAA Board of Scientific Counselors, which comprises eminent senior scientists from outside NIH, comprehensively reviews each laboratory’s ongoing research at least once every 4 years. The Board critically evaluates each intramural laboratory’s current research, offers suggestions for improving research, makes recommendations on the promotion and tenure of individual scientists, and proposes promising areas for expanded research efforts. Reports containing these evaluations, and the actions taken in response to them, are then summarized for the NIH Board of Scientific Directors, who direct the intramural programs of all the NIH Institutes and for the NIAAA National Advisory Council, which oversees the quality of NIAAA’s overall program.

Because of the retrospective review process, the IRP can change its research priorities more expeditiously than can most participants in the prospectively reviewed ERP. Thus, the IRP has the unique flexibility to pursue promising new avenues of inquiry. In addition, intramural research funding and other resources are generally stable over relatively long periods of time, whereas extramural grants must be applied for at regular intervals. Stable funding enables the IRP to conduct long-term research projects that may have uncertain outcomes, capitalizing on the equipment and expertise available at NIH.

## Basic Research in the IRP

The genetic and nongenetic research conducted in the IRP share several common themes. These include the molecular theme of identifying alterations in serotonin^1^ function in brain cells of individuals at risk for alcoholism; the clinical theme of classifying subtypes of alcoholic individuals by characteristics such as age at disease onset and associated psychiatric and behavioral problems; and the neurophysiological theme of focusing on trait differences, including inherited variations in people’s electroencephalograms (EEG’s) and differences in their neuroendocrine and behavioral responses made in the laboratory under the influence of specific pharmacological agents. Each theme is discussed below.

### Genetic Research

Researchers have long known that people who have parents or other close relatives with alcohol problems are more likely to develop similar problems than are those who do not have this family history, thereby implicating a genetic element in alcoholism. Studies with twins have reinforced this idea by providing clear evidence that genetic factors play an important role in an individual’s vulnerability to alcoholism. This observation has prompted scientists to explore the genetics of alcoholism, because identifying genetic factors would likely lead to better prevention and treatment of the disease. Alcoholics may respond differently to different treatments depending on the factors that cause their drinking problems. Scientists may be able to design new treatments for particular groups of alcoholics based on an understanding of the various biological and environmental factors contributing to their alcohol problems.

The increased risk of developing alcohol problems that are associated with being the child of an alcoholic does not mean that this outcome is inevitable. Both genetic and environmental factors undoubtedly influence whether alcohol problems develop. For example, people who have inherited either impulsive or anxious temperaments are more likely to become alcoholic, but only a minority do. Identifying genetic vulnerability factors helps to reveal the impact of environmental influences, which interact with the genetic factors in complex ways. Whatever the ultimate nature of the genetic factors involved in alcoholism, they almost certainly are complex, involve multiple genes, and interact with early developmental experiences as well as current life stresses. A significant part of the IRP’s genetic research effort focuses on characterizing these factors and the relationships between them.

#### Population Studies

The IRP projects aimed at solving the puzzles presented by the genetics of alcoholism cover a range of topics. Researchers in the Laboratory of Neurogenetics (LN) are studying alcoholism in particularly high-risk, homogeneous groups, including large American Indian families. Because alcoholism is highly prevalent in many American Indian communities, it is particularly important for researchers to define the role of genetic vulnerability factors and such interacting environmental factors as employment, income, acculturation, and education in these populations. In studies on American Indians and other groups that are genetically, socially, and clinically more homogeneous than the general population, researchers also seek to isolate genetic elements that can be associated more widely with the general population’s vulnerability to alcohol problems.

#### Molecular Studies

**Figure 2:**
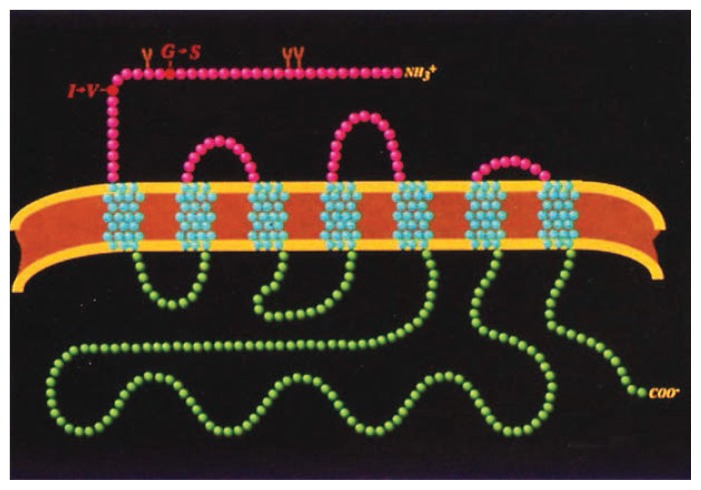
The structure of a serotonin receptor subtype (a protein that binds the neurotransmitter serotonin in the nervous system). Two amino acid substitutions (amino acids are the building blocks of proteins) have been found to occur in rare instances in this subtype. The substitutions are the result of differences in the genes that encode the protein. In the first, Glycine (G) is substituted for Serine (S). In the second, Isoleucine (I) is substituted for Valine (V). Studies of these receptor differences, which exist in some people but not everyone, could lead to the identification of differences in receptor function that influence alcohol preference and impulsive behavior.

In another project, LN scientists are directly examining gene structure, revealing differences in the makeup of key proteins involved in signal transmission among nerve cells. For example, LN scientists have found variations of several of the proteins involved in the function of the neurotransmitter serotonin among subjects and are now investigating these variations for their possible involvement in vulnerability to alcoholism (figure 2).

LN scientists also are investigating the control of the expression of genes that are involved in the function of neurotransmitters. This research is important because alcohol itself, withdrawal from alcohol, and other stressors can alter gene expression and actually change the way the brain works. For example, serotonin ordinarily inhibits a variety of behaviors, including aggression, food consumption, and probably alcohol consumption. However, certain stresses can reverse this inhibition. LN scientists recently have identified regulatory elements at the gene for tryptophan hydroxylase, a critical enzyme involved in serotonin synthesis. Activation of these elements—by, for example, glycocorticoid stress hormones—produces profound changes in the expression of tryptophan hydroxylase, which in turn alters serotonin synthesis. Differences in serotonin levels predispose some people to more impulsive behaviors, including alcoholism. Therefore, scientists must identify the genetic variants that determine differences in the genes involved in the function of this neurotransmitter as well as determine the molecular mechanisms by which environmental factors can exert an influence, such as changing serotonin’s rate of synthesis.

### Molecular and Cellular Research

Much of the IRP’s basic research focuses on understanding alcohol’s effects on the body at the most basic cellular and molecular levels. Research includes work by the Laboratory of Molecular and Cellular Neurobiology (LMCN) in determining alcohol’s effects on certain neurotransmitters, such as serotonin (discussed above), and their receptors, which are fundamental to the various communications occurring within and between brain cells and which alcohol appears to disrupt. Other research conducted by the Laboratory of Membrane Biochemistry and Biophysics (LMBB) includes studies of cell membrane structure and function and how alcohol affects essential membrane structural components called fatty acids.

#### Alcohol’s Effects on Neurotransmitter Systems

Receptors are proteins in the cell membrane that react to specific neurotransmitters normally present in the brain, such as serotonin. However, receptors also respond to the disruptive effects of alcohol and other drugs. LMCN scientists were the first to discover that alcohol specifically affects a receptor for the neurotransmitter glutamate, the NMDA receptor (named for N-methyl-D-aspartate), which is known to be involved in cognitive activity and motor coordination.

When binding to the NMDA receptor, glutamate activates a system permitting calcium to enter the nerve cell, which in turn triggers other cellular processes that excite the nerve cell and produce nerve impulses.

The LMCN studies showed that alcohol’s effect on the receptor, which inhibits the nerve cell from sending impulses, depends on concentrations in the range of intoxicating levels, making it a likely candidate for involvement in the behavioral effects of intoxication. Because the NMDA receptor also is known to be involved in learning, early fetal development, and susceptibility to seizures, such a common mechanism could well explain alcohol’s diverse effects on memory and fetal development and possibly could explain physical dependence and symptoms such as seizures that often result from alcohol withdrawal.

It is important to recognize that the many transmitter systems in the brain, such as the NMDA/glutamate system, do not act in isolation. Alcohol’s actions on one system may trigger events in other regulatory systems or change the sensitivity of those systems to their normal stimuli.

Current work by LMCN scientists on the NMDA receptor and several other neurotransmitter receptors is in the vanguard of basic alcohol research. Although most of this work has concentrated on immediate alcohol effects, studies of the long-term effects of alcohol on neuroreceptor systems also are in progress. Such effects include changes in receptor gene regulation and expression (discussed above), changes in the structure of receptors, and modified nerve cell development in fetal alcohol syndrome (FAS). LMCN’s work also includes research on the molecular and cellular basis of other behavioral effects of alcoholism, such as tolerance (the requirement, over time, for increased amounts of alcohol to produce the same effect) and craving.

#### Alcohol’s Effects on Cell Membranes

In other studies of molecular and cellular biology, LMBB has made major advances in understanding alcohol’s effects on fatty acids in cell membranes. These fatty acids are found in the form of complex phospholipids and play an essential role in the function of cell membranes. Chronic alcohol consumption leads to a decrease in the levels of essential fatty acids in various organs, including the brain. In addition, an inadequate supply of fatty acids during fetal development (if the mother’s diet is deficient) and during postnatal early development leads to a deficit in cognitive and visual functions. These deficits may occur because repeated alcohol intake, combined with an inadequate diet, prevents the accumulation of fatty acids in the fetus’s brain. Researchers believe that the alcohol-induced decline (or insufficient buildup in fetuses) of particular fatty acids in the nervous system underlies some of the pathological effects of alcohol abuse on the brain.

Taking this research one step further to investigate how alcohol causes fatty acid levels to decline, recent studies have suggested that alcohol can alter the metabolism of these membrane lipids so that they are degraded faster by the body’s natural processes. It appears that the brain or liver or both attempt to compensate for this increased rate of degradation by increasing the rate at which essential fatty acids are synthesized. However, when the alcohol dose is too high or repeated too often (as it is in alcoholism), the system cannot compensate, and the level of essential fatty acids may become inadequate for the cells to function properly. A poor diet (i.e., one that has low levels of essential fatty acids and antioxidant vitamins and minerals) may exacerbate this effect. Many alcoholics obtain much of their caloric intake from alcohol and do not have the benefit of a varied, nutritious diet.

**The History of Intramural Research at NIAAA**

**Figure:**
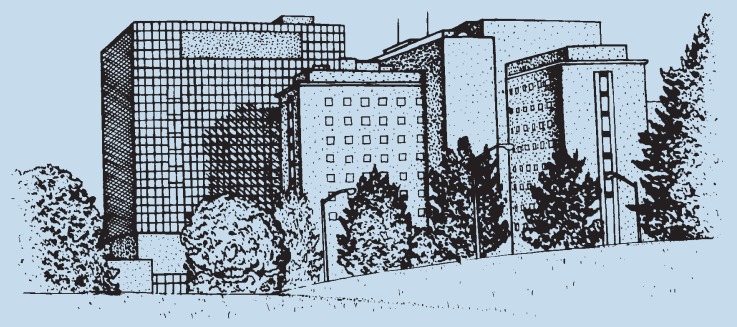
Ultimately the Intramural Research Program moved to Building 10 at the National Institutes of Health in Bethesda, MD.

In 1966 the prevailing view of alcoholism as a breach of morality began to give way to the perception of the disorder as an addiction, a condition at least partially beyond the control of its sufferers. The public and, more importantly, physicians and scientists began to accept that alcoholism might be a medical problem, a disease brought on by complex factors that works insidiously to destroy a range of body functions, from liver and pancreatic activity to mental processes, such as memory.

Prior to this period, the Federal Government had provided little funding to support alcoholism research. In October 1966, however, the National Institute of Mental Health (NIMH), directed by Stanley F. Yolles, M.D., established the National Center for the Prevention and Control of Alcoholism. The new Center, which was located with NIMH at St. Elizabeths Hospital in Washington, DC, was intended to support research endeavors and efforts to train scientists in the alcoholism field. It also was responsible for communicating research findings to educate the public and to better inform people in the treatment and service arenas of the field. Yolles recruited Jack H. Mendelson, M.D., an alcoholism researcher at Harvard University, as the Center’s first director (Mello and Mendelson 1971).

***Setting Up: The First Years***

Before he would agree to direct the Center, Mendelson insisted that in addition to promoting extramural research (by providing grants to applicants from private institutions wishing to study aspects of alcoholism), the Center initiate an Intramural Research Program (IRP) to be run by Center scientists at laboratory facilities at St. Elizabeths. NIMH agreed to support intramural research, and Nancy K. Mello, Ph.D., also from Harvard University, was appointed as the IRP director.

Both Mendelson and Mello first became committed to understanding alcoholism and its causes while receiving their medical and scientific training in Boston, MA. According to Mendelson, at that time both researchers “were impressed with [the prevalence of] a number of biomedical problems” among patients at the city’s hospitals. “One of the major ones was alcohol abuse and dependence,” he said.

The two researchers believed that alcoholism should be an area of mutual interest for scientists trained in a range of fields, such as cardiology and molecular biology as well as psychology. According to Mendelson, they believed an intramural program could “maintain continuity for multidisciplinary investigative work,” allowing long-term, cross-disciplinary studies to be conducted without interruption. The program also could “provide a very important resource with respect to training individuals from various disciplines” to do alcoholism research.

With the goal of attracting scientists and postdoctoral research fellows with a variety of backgrounds, the Center began recruiting. The directors found that despite the widespread stigma still attached to alcoholism, scientists wanted to carry out research in this field. As a result of these efforts, research projects progressed over the next several years at St. Elizabeths in areas such as clinical investigations of pharmacological treatments for alcohol craving and withdrawal syndrome; genetic predisposition to alcoholism; alcohol metabolism; alcohol’s effects on the brain; and the design and breeding of animal models of alcohol dependence, including some of the first models of alcoholism in other primates (i.e., rhesus monkeys).

According to Mendelson, the IRP was, and still is, unique in its ability to “bring persons together to work in concert” who possess different fields of knowledge and varying ideas about how alcohol may effect disease, encouraging people to “share their ideas and interact” to achieve results that “hopefully transcend what their own special interests are.”

***Gaining Acceptance: 1978–82***

Research continued in both the extramural and intramural programs after the Center became the National Institute on Alcohol Abuse and Alcoholism (NIAAA) in 1971 (see the article by Hewitt, pp. 12–16). A handful of researchers affiliated with the IRP, however, believed that it would not be recognized as a center of leading-edge research until it could be located with other biomedical intramural programs on the campus of the National Institutes of Health (NIH) in Bethesda, MD. The old stigma dismissing alcoholism as a mental problem that plagued only social deviants still echoed through the research community, making it difficult for alcoholism researchers to have their work acknowledged as a contribution to medicine.

In 1977 NIAAA asked Gian C. Salmoiraghi, M.D., Ph.D., a former director of NIMH’s Division of Special Mental Health Research, of the Clinical Neuropharmacology Research Center, and of research at St. Elizabeths Hospital, to become the IRP’s acting director. Salmoiraghi believed that the program “needed to acquire legitimacy for the field” by being accepted as a part of the larger intramural research community at NIH. He met with NIH’s director of intramural research, DeWitt Stetten, M.D., to press his case. By pointing out to Stetten that, according to national statistics, 10 percent of the patients admitted to NIH’s clinical wards had alcohol problems that should be understood and addressed, Salmoiraghi “achieved tolerance at NIH.” The IRP was promised space for housing patients and conducting clinical research in NIH’s clinical building on the Bethesda campus.

During his tenure as acting IRP director, Salmoiraghi recruited Markku Linnoila, M.D., Ph.D., who now directs the IRP, as chief of the newly created clinical laboratory at NIH. Moving the IRP to NIH would allow collaborations to develop, not only between scientists of various disciplines but between intramural programs as well. The extensive facilities and other opportunities the IRP found at NIH eventually would allow the program to broaden its research focus and grow to become the multidisciplinary center envisioned by its former directors.

***Expansion: 1984–90***

When Boris Tabakoff, Ph.D., took over as director of the IRP in 1984, its clinical laboratory still was being organized on the NIH campus, and according to Tabakoff, the program was drawing from its new location “tremendous leverage” to become like the other highly diversified IRP’s at NIH. For example, unlike private hospitals, which are hampered by costs, the clinical laboratory could house 10 patients on its ward for at least 40 to 50 days, allowing physicians to observe phenomena that appear over time, such as the effects of detoxification and withdrawal on memory.

Also, NIAAA researchers could gain access to new technologies, such as costly imaging equipment, by sharing the expense with other NIH IRP’s and recruiting imaging experts who merged their knowledge with that of the alcoholism researchers to develop new insights and novel techniques for use in future studies. Thus, the IRP pioneered research in certain fields, as it has in its ongoing use of magnetic resonance imaging (MRI) to study alcohol’s damaging effects on brain function.

Because of the quality of its research and its leading-edge direction, the program continued to gain acceptance in the NIH community during the mid-1980’s. During his tenure as the IRP director, Tabakoff also was invited to represent NIAAA on NIH’s decisionmaking body, the Board of Scientific Directors, something NIAAA had been excluded from when he arrived. This accomplishment symbolizes what Tabakoff regarded as the “complete turnaround” in NIH’s attitude toward research on alcoholism that had taken place since the IRP’s founding at St. Elizabeths Hospital.

As the IRP received more space and respect at NIH, it expanded its research efforts in areas such as genetics; molecular biology (i.e., the study of the alcohol molecule’s effects on the human body’s chemistry); immunology, including alcohol and AIDS; imaging; and the efficacy of new medications in treating aspects of alcoholism. It also undertook research collaborations with other IRP’s, such as those of the National Cancer Institute and the National Institute of Diabetes and Digestive and Kidney Diseases. The training program for physicians and Ph.D. researchers also continued to expand and diversify, enlisting specialists from internists and psychiatrists to molecular geneticists to conduct clinical and basic research while learning about the alcoholism field (Tabakoff and Petersen 1988). The IRP’s access to NIH facilities and its unique regimen of research projects attracted many of these trainees.

Tabakoff noted that under his direction, the IRP made initial contributions to alcohol research in areas such as neurology and immunology that later were carried on by private institutions through the extramural program. Such interplay allowed the two programs to complement, rather than compete with, each other.

Like his predecessors, Tabakoff emphasized certain unique aspects of the IRP in speaking about its value to the field of alcoholism research. One aspect is the program’s flexibility. “Researchers,” he said, “do not need to achieve results within a specified length of time, allowing for the pursuit of projects that have a long period before payoff.” But the same flexibility allows IRP scientists to shift their priorities to take advantage of “windows of opportunity” for new research. Two such projects began when the need to study relationships between alcohol and AIDS became apparent and when the value of MRI in studying brain function was demonstrated.

From its inception, NIAAA’s IRP has been intended to conduct leading-edge projects in many areas of biomedical and psychological research. The IRP has helped to tear down barriers, such as the stigma surrounding alcoholism; has reached its goal of becoming a multidisciplinary program; and, according to Mendelson, has “maintained throughout the years a tradition of excellence.”

—Kathryn G. Ingle

Kathryn G. Ingle, M.A., is a science editor of Alcohol Health & Research World.

ReferencesMelloNKMendelsonJHRecent Advances in Studies of Alcoholism: An Interdisciplinary SymposiumNational Institute of Mental Health (NIMH) Pub. No. (HSM)71–9045Rockville, MDNIMH1971TabakoffBPetersenRCReports from research centers–13: Intramural research program of the National Institute on Alcohol Abuse and AlcoholismBritish Journal of Addiction8354955041988283811610.1111/j.1360-0443.1988.tb02568.x

#### Alcohol’s Actions on Membrane Components

Another example of basic research conducted on the molecular level involves alcohol’s direct effects on the molecules composing the cell membrane. LMBB researchers have shown that alcohol interacts with membrane lipids at the cell’s surface in much the same way that water does. Under normal conditions, water covers the entire membrane surface, including the lipids, proteins, and carbohydrates. The layer of water around these molecules is important for maintaining their conformations (i.e., their size and structure) and functions, and it is very likely that the replacement of water by alcohol in this layer alters these functions.

Researchers also have found that although alcohol acts on the membrane surface, the results of the interaction affect the membrane’s interior. Changes caused by alcohol at both the surface and interior of the cell membrane can alter the cell’s function. To study alcohol’s influence on the membrane’s interior, LMBB has modeled the outside of the cell where alcohol acts with an artificial membrane using the lipid components most commonly found in the outer membrane leaflet. For the first time, researchers have detailed the effect of each of these components on the way in which alcohol alters membrane structure at all depths across the membrane, including the interior. These studies are important in understanding how alcohol affects membranes in living cells.

**Figure 3:**
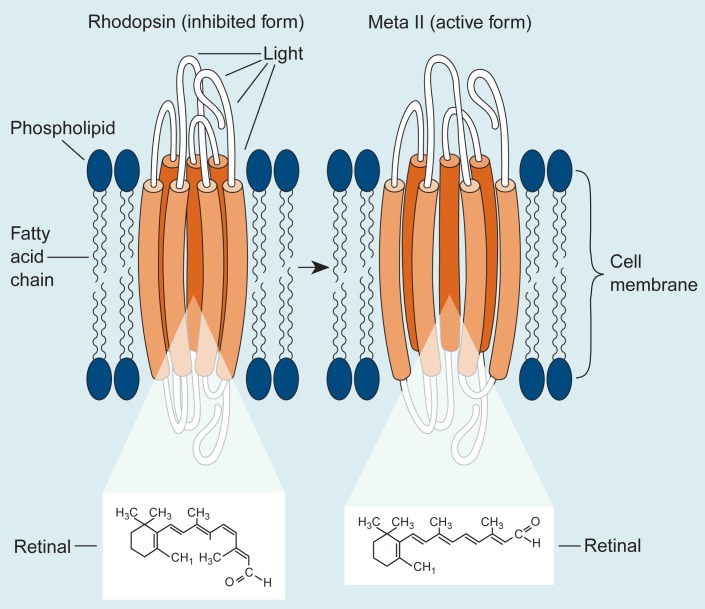
The structure of the protein rhodopsin, a typical G protein-coupled receptor, and its activated form, metarhodopsin II (Meta II), located in a cell membrane. The Meta II form is created when a molecule at its center, called retinal, changes its conformation in response to absorbing light. When retinal changes, the receptor increases in volume. Alcohol increases the amount of active receptor (i.e., Meta II) formed by loosening the packing of the phospholipid fatty acids in the membrane surrounding rhodopsin and allowing it to expand into its Meta II conformation. Neuronal and retinal tissues are rich in the specific phospholipid fatty acids that respond to alcohol’s presence, which suggests that the phospholipids may play a role in modifying receptor activity in response to acute alcohol exposure.

One example of this type of research in LMBB is the first direct observation that alcohol’s ability to change membrane receptor function can depend on its effects on the lipid content of the membrane surrounding the receptor. Scientists examined alcohol’s effect on the function of the visual receptor, rhodopsin, in cell membranes taken from the retina of the eye. Different from the receptors previously described, this G protein-coupled receptor system is responsible for low-light-level vision. When rhodopsin absorbs light, it activates a G protein in the cell’s interior by increasing in volume to assume its activated form, called metarhodopsin II (or Meta II). Researchers found that alcohol loosens the packed phospholipid fatty acids (i.e., the portion of the lipids that makes up the membrane’s interior) around a rhodopsin molecule, enhancing the receptor’s ability to expand into its Meta II conformation (figure 3). When researchers replaced the lipids in their model membranes with polyunsaturated fatty acids, alcohol’s effect on phospholipid packing was even more pronounced.

The increased formation of Meta II produced by short-term alcohol exposure probably results in the hyperactivity of this signaling pathway. In contrast to this finding, the previously described loss of polyunsaturated fatty acid components of membrane phospholipids caused by long-term exposure to alcohol may diminish alcohol’s effect on this pathway. Hence, the loss of polyunsaturated fatty acids may contribute to alcohol tolerance developed during chronic alcohol use. These findings also agree with the observed impairment of certain visual functions previously described as related to a deficiency in essential fatty acids during fetal development.

#### Alcohol’s Effects on Fetal Development

The fetal effects of heavy maternal alcohol use also are well known, but again, their cellular and molecular bases are not. LMBB is developing a new animal model of FAS to enable further study of the damage alcohol inflicts during fetal development. The model is based on the theory (discussed above) that alcohol prevents fatty acids from accumulating in the brain, thereby contributing to developmental difficulties. The combination of a diet limited in polyunsaturated fats (of which fish is a good source) together with daily alcohol exposure during pregnancy may enhance alcohol’s negative effect on fatty acid buildup. Scientists are investigating the combination of alcohol and this type of diet in animals as an appropriate model of those alcoholics, including some pregnant women, who experience periods of poor nutrition. Thus, the offspring of animals maintained on this diet and given alcohol may reflect more accurately the molecular characteristics of a person with alcohol-related birth defects than have past models.

Other laboratory studies conducted within LMCN use in vitro (i.e., simple controlled cell cultures) or in vivo (i.e., slices of rat brain) models to study the molecular basis of alcohol’s neurotoxicity and its effects on the formation of connections between cells in the developing brain. These studies will show how alcohol disrupts the development of brain cells and the communication paths between cells and also will help explain the physiological damage seen in FAS.

#### Animal Model Development

One great difficulty encountered in research—even with the newer neuroimaging techniques, such as positron emission tomography (PET) and magnetic resonance imaging (MRI), that are now available for studying the intact human brain—has been the investigation of alcohol’s action at molecular and cellular levels. Therefore, an important aspect of intramural research has involved developing appropriate animal models for use in many areas in addition to the developmental studies mentioned above. These include using nonhuman primates to elucidate the molecular, genetic, developmental, and environmental variables contributing to individual differences in alcohol preference, sensitivity, and consumption levels.

## Clinical Studies

Although basic research on the underlying mechanisms involved in alcohol abuse and alcoholism is crucial to devising rational prevention and treatment strategies, these clinical goals also must be pursued directly. The IRP’s clinical research efforts are directed toward defining more accurately alcohol’s adverse health consequences, making diagnostic techniques more precise and less dependent on patients’ own descriptions of their drinking behavior, and devising improved interventions. Ultimately, alcohol abuse and alcoholism are human problems that must be faced in the very real world of the family, the school, the workplace, and the clinic.

Although most animals can be induced to consume alcohol, they typically do not abuse it in the same way that people do, which limits their usefulness as models of alcoholism. Moreover, animals are not affected by the cultural, social, intrapsychic, and interpersonal forces that play roles in alcohol use by humans. Psychosocial and biological differences between humans and animals make human and animal research complementary; neither can adequately replace the other.

Clinical studies currently underway at the IRP are designed to explore many aspects of alcoholism that parallel those being studied in the basic research laboratories—from alcoholism’s genetic determinants and biological origins, and its impact on neuroanatomy and cognitive functioning, to improved intervention methods. Investigators are pursuing clinical research on these aspects of alcoholism in populations ranging from alcoholic patients and their families living in the Washington, DC, area and American Indians on reservations in the Southwest to violence-prone male alcoholics living in Finland (mentioned above).

### Clinical Consideration of Genetics

Investigations of the genetic and biological bases for alcoholism involve examining families in which alcoholism is present. By studying families with an alcoholic member, researchers can look for shared causal factors, ranging from the family’s dynamics to members having similar genetic and neurophysiological characteristics. Studying biological similarities between alcoholic patients and their children may reveal characteristics that could lead to the early detection of children at risk for abusing alcohol. Intramural research in this area potentially can identify biological indicators, or “markers,” of vulnerability to alcohol abuse. The indicators (i.e., observable characteristics) are known as markers if they reliably are found to be inherited along with a tendency to develop alcoholism. Such markers eventually may be useful in detecting vulnerability to alcohol’s effects even before a person takes his or her first drink.

One example of a potential biological marker for alcoholism is the work done by the Laboratory of Clinical Studies (LCS) on examining characteristic patterns of the brain’s electrical activity (referred to as EEG activity). The distinct patterns found in certain individuals may be useful as markers of susceptibility to alcohol problems. In addition, the ways in which the EEG patterns are transmitted genetically within families could provide clues to the ways in which alcoholism itself is inherited.

### Physiological Aspects of Clinical Research

#### Metabolism and Nutrition

One example of the shared interests of basic and clinical research focuses again on alcohol’s interaction with fatty acids. IRP researchers in LMBB are studying the synthesis and breakdown of essential fatty acids by the liver in both adults and infants. The researchers are using a highly sensitive and specific, noninvasive technique, developed in LMBB, that measures the liver’s metabolic activity with respect to converting dietary fatty acids to the more saturated forms required by the brain and other organs. This study may help scientists understand the mechanism that underlies the loss of essential fatty acids in alcoholics.

In related research, scientists studied premature and term infants to determine the stage of maturity at which the liver is capable of forming the essential fatty acids required for optimal brain development. A surprising result of this study was that even quite premature infants had the capacity to convert these fatty acids in the liver within 1 week after birth. Basic studies such as this are crucial for developing an understanding of the pathways by which the nervous system accumulates the specific polyunsaturated fatty acids that it requires and the disruption of this process during fetal alcohol exposure. Furthermore, this study has potentially important implications for the formulation of infant formulas that optimally support neural development.

#### Imaging

**Figure 4:**
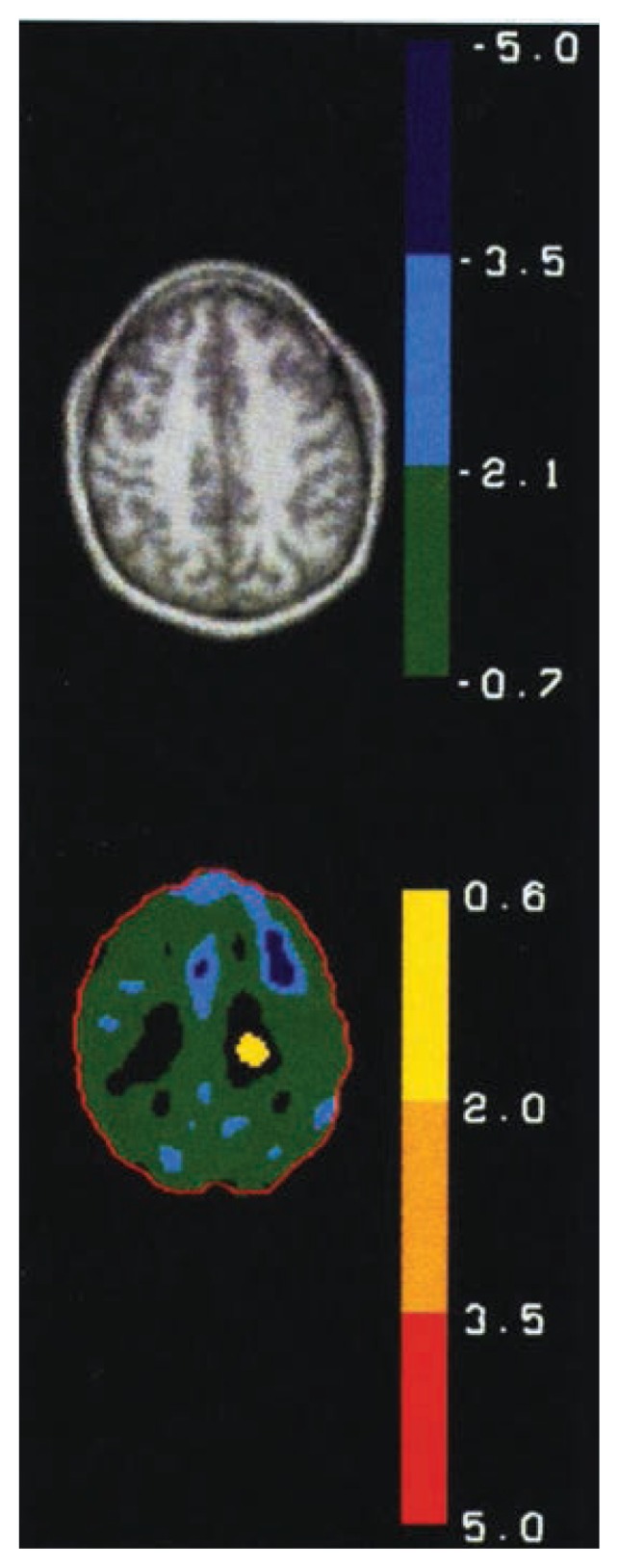
An axial slice (i.e., an image taken along a horizontal plane) from a magnetic resonance imaging-generated picture of the brain. In the lower image, the same slice is shown as a compilation taken from positron emission tomography (PET) scans of 19 detoxified alcoholics and 10 nonalcoholic volunteer males. The PET image shows differences between the alcoholics and the volunteers in the brain’s glucose metabolism. The dark blue regions are areas in which the alcoholics exhibited significantly lower glucose use than the volunteers.

Advanced brain imaging techniques—PET and MRI—increasingly have served as important noninvasive tools for studying the living human brain. These techniques permit researchers to detect more accurately neuroanatomical changes correlated with chronic alcohol use and functional changes in brain tissue, such as rates of glucose metabolism, which is the body’s major energy source. LCS researchers compare images of a person over time or of alcoholics with nonalcoholics and quantify regional changes or differences in the brain (figure 4).

Scientists have criticized imaging studies, however, for their lack of an acceptable means of determining the significance, in statistical terms, of differences in brain images obtained by MRI and PET scanning. Recently LCS researchers have made progress in this area using sophisticated statistical techniques for analyzing these complex images. Because determining whether differences between brain images are significant is such a basic problem with this otherwise useful tool, LCS also is pursuing alternative ways of determining these differences.

Using the recently developed statistical techniques, researchers have found correlations among regional brain glucose metabolism and age, severity of alcoholism, and cognitive ability. Brain glucose metabolism tends to decrease (i.e., is negatively correlated) with advancing age in alcoholics but not in nonalcoholics. PET scans of patients who show loss of control and are physically violent also have shown significant decreases in the patients’ ability to metabolize glucose in certain brain areas. Such patients also experience a greater frequency of panic attacks, alcohol abuse, and alcoholism than do healthy volunteers.

Researchers have used another imaging medium—MRI—since early 1994 to study olfactory stimulation’s effects on regional brain blood flow. The goal of this research is to understand the functional neuroanatomy of brain areas associated with the desire for food or alcohol. Thus far, researchers have found that pleasant odors produce a significant increase in blood flow in specific brain areas—the nucleus accumbens, which includes an olfactory center, and the amygdala and hypothalamus, both of which are involved in behavioral functioning. This research may help scientists devise better ways of reducing drinking relapses in alcoholics undergoing treatment by investigating alcoholics’ brain responses to alcoholic beverages.

Intramural neuroimaging research also has emphasized the use of neuroimaging methods that can provide detailed three-dimensional views of the living human brain. Such views enable scientists to visualize and measure more accurately neuroanatomical and functional changes associated with heavy alcohol use.

#### Pharmacological-Challenge Research

Among the most serious effects of chronic heavy drinking are those on thinking, problem-solving, and memory. Unlike nonalcoholic volunteers, the aging, or neuropsychiatrically impaired groups, alcoholics show selective impairments in those cognitive functions involved in monitoring mental operations under voluntary control. Drug challenges (i.e., the acute administration to nonalcoholics of drugs other than alcohol that mimic some of alcohol’s effects) are useful in delineating the types of impairment that are present in alcoholics and others. Because the actions of the drugs being given to subjects are well defined, researchers can clarify alcohol’s effects by determining whether it produces the same reactions in the subjects as the other drugs produce. For example, acute doses of benzodiazepine tranquilizers such as diazepam mimic some of alcohol’s effects on cognition, such as a reduced ability to judge one’s own accuracy of memory recall and of identifying sources of the information that is remembered. These drug-induced deficiencies also resemble those produced by certain anesthetics such as ketamine. Ketamine is a known blocker (i.e., antagonist) of the receptor believed to be involved in long-term memory consolidation. Certain drugs—benzodiazepines and alcohol—administered to normal subjects also produce changes in how they, as well as how alcoholics, retrieve information. Thus, these drugs may act on the same brain receptor as ketamine.

The effects of these drugs on certain aspects of memory may be important in understanding patterns of uncontrolled drinking, alcohol’s reinforcing or rewarding effects, and the reduced ability of alcoholics to recognize various stimuli. The IRP is now extending this research to study alcohol craving and cognitive functions in children at risk for developing alcohol-related problems.

Other pharmacological-challenge research is helping to clarify the distinction between early onset (i.e., type II—younger than age 25) and late onset (i.e., type I—older than age 25) alcoholism. When a drug that mimics certain effects of the neurotransmitter serotonin, called m-chlorophenyl-piperazine (m-CPP), was given to detoxified alcoholics, it led to alcohol craving in many early onset alcoholics but to heightened anxiety, a very different effect, in late onset alcoholics. This outcome implies that a physiological difference in certain serotonin functions exists between the two types of alcoholics. A possible difference in serotonergic function in the two groups also is suggested by the fact that early onset alcoholics also have relatively lower levels of serotonin activity in the brain. Early onset alcoholics may have normal levels of serotonin while drinking, although many of them have abnormally low levels when abstinent. Results from similar challenge paradigms suggest that numerous biological differences may exist between alcoholics and nonalcoholics. Thus, in some patients, drinking may represent a form of self-medication, in which alcohol is used to raise inherently low levels of serotonin or other important chemicals.

Pharmacological-challenge research also has demonstrated that the brain undergoes significant adaptive changes as a result of long-term, excessive alcohol consumption. Administration of sodium lactate causes panic in individuals who have panic disorder. But when it is given to detoxified alcoholics diagnosed as having panic disorder, it results in a lower frequency of panic attacks than it does in nonalcoholic patients with panic disorder. Such adaptive changes undoubtedly play a role in alcohol tolerance as well as determine the withdrawal symptoms that ensue when drinking is stopped.

## Prevention and Treatment

### Techniques for Prevention

The IRP’s ultimate objective is not only to understand the mechanisms governing alcohol’s effects but also to employ this knowledge for better prevention and treatment of alcohol abuse’s effects. For example, if some of alcohol’s deleterious effects, such as those on the liver and nervous system, are related to reduced levels of polyunsaturated fatty acids (as discussed earlier), simple strategies for preventing or treating these complications may be to avert these losses or to restore the proper levels. Modern nutritional research already has described diets to accomplish these ends for both the liver and the peripheral nervous system. Some success has been achieved in preventing the alcohol-induced losses in liver lipids during continuous alcohol exposure. The brain is more refractory to dietary manipulation, and in fact, alcohol is one of the few biochemical or nutritional means of altering neural fatty acid levels. It is still unknown whether the strategy of altering diet can be applied successfully to prevent some of the neuropathological and central nervous system functional deficits caused by alcohol. If such diets were even partially successful, they would represent a simple and inexpensive way to help prevent some of alcohol’s damaging effects on both adult alcoholics and their children (e.g., alcohol-related birth defects).

### Medications That Assist in Treatment

The use of medications to assist alcoholics in remaining abstinent has a long history. Disulfiram, better known by the trade name Antabuse^®^, is probably the most widely used drug for this purpose. However, it is somewhat effective only in alcoholics who are highly motivated to avoid drinking, and it may cause serious side effects. Should alcohol be deliberately or inadvertently consumed after ingesting disulfiram, the body’s reactions range from acutely unpleasant flushing and nausea to life-threatening responses, depending on the amount ingested and the individual’s sensitivity to the drug’s effects. The IRP’s continuing goal is to search for medications that can specifically block alcohol’s effects, reduce its reinforcing properties, or otherwise alter the impulse to drink without having seriously toxic effects.

## Future Directions

If earlier research productivity is any indication, the IRP’s future is likely to be as impressive as its past. In some areas, intramural scientists have pioneered important research, including the role of NMDA neurotransmitter receptors in alcohol intoxication and dependence and more recently the role of other receptors (e.g., 5-hydroxytryptamine-3 [5-HT-3] and adenosine triphosphate) in the brain in producing alcohol’s effects. For example, the IRP is one of the few alcohol research programs in the world fully capable of exploiting new molecular neurobiological advances in receptor research.

### Polymorphic Gene Research

Certain PET imaging studies are important to the further exploration of the brain function of polymorphic genes (i.e., genes with multiple forms that vary among individuals) that are thought to be involved in alcoholism susceptibility. These PET studies employ chemical compounds (which are injected) that are capable of tagging proteins encoded by the genes so that the proteins appear lit up on the image. Another important step in polymorphic gene research involves determining the consequences to a person’s physiological and behavioral functioning of expressing certain gene forms as proteins.

The IRP also is pursuing the technical objective of inserting polymorphic genes into expression systems, such as frog eggs, to investigate the genes’ molecular and cellular physiology. This work is essential in determining which genes are associated with alcoholism; how variations in those genes, or their expression, produce susceptibility; and how cellular functions are changed by the products that result from gene variability.

### Animal Models Research

In the animal research area, investigators are planning studies of transgenic animals, which carry human deoxyribonucleic acid (DNA). This will allow detailed studies of human genetics in an animal model. Similarly, selectively breeding monkeys for their vulnerability to alcohol abuse and for poor control of behavioral impulsiveness may provide researchers with a useful animal model of human alcoholic behavior.

## Conclusions

As in other health areas, basic alcohol research is likely to yield significant benefits that are difficult to predict in advance. If the history of science demonstrates anything, it is that research on the basic mechanisms underlying outcomes is more likely to produce important breakthroughs than is more narrowly defined applied research. Both types of research, however, clearly are needed because they complement each other. The continuing task of NIAAA’s intramural scientists is to pursue research of the highest quality in both basic and applied areas and to provide leadership in alcohol research in ways that only such a diverse program with a critical mass of expertise can.

## Footnotes

1 For definitions of this and other technical terms used in this article, see glossary, p. 67.

## Glossary

Alcohol abuse: Refers to patterns of problem drinking that have resulted in detrimental effects on a person’s health and/or social interactions.
Alcohol dependence: Also known as alcoholism. Refers to a disease characterized by abnormal alcohol-seeking behavior that leads to impaired control over drinking.
Antioxidant: A substance that inhibits chemical oxidation (a chemical reaction that usually involves loss of hydrogen from a molecule), such as vitamin E.
Electroencephalogram (EEG): A recording of electrical activity that emanates spontaneously from nerve cells in the brain. Variations in the patterns of an EEG correlate well with activity and conditions in the brain. Thus, EEG’s often are used as diagnostic tools.
Fatty acids: Molecules that constitute components of fats (i.e., lipids). Fatty acids are essential components of the *phospholipids* in cell membranes. Saturated fatty acids differ structurally from polyunsaturated ones in the numbers of double-bonds that exist between the carbon atoms that make up their chains. Saturated fatty acids have no double bonds and are less likely to unite with other compounds. Polyunsaturated fatty acids usually have between three and six double bonds and are more reactive.
G proteins: Molecules within cells that act as messengers, linking protein receptors in the membrane to molecules that perform specific functions inside the cells (e.g., enzymes, which are proteins that accelerate specific chemical reactions).
Gene: A sequence of nucleotides (i.e., the basic molecular units of deoxyribonucleic acid [DNA]) that, when translated by mechanisms in a cell, encode a protein.
Gene regulation: The process of modifying the rate at which a *gene* is expressed as a protein. Sequences of DNA that are called *regulatory elements* influence *gene* expression.
Neuroendocrine: Refers to interactions between the nervous and endocrine systems of the body. For example, nerve cell activity often is influenced by hormones secreted by endocrine glands, such as the pituitary.
Neurotransmitters: Members of a group of substances in the nervous system that are responsible for transferring a nerve impulse from one nerve cell (i.e., neuron) to the next. These chemicals are released by one neuron and travel to a target neuron. At the target neuron’s surface, they attach, or bind, to specific receptors to produce an impulse in the target neuron.
Neurotransmitter receptors: Proteins located on the outer surface of neurons that attach, or bind, specific *neurotransmitters* to become activated and either initiate or inhibit nerve impulses. These receptors (e.g., the NMDA receptor) often are named for synthesized compounds that they bind in addition to naturally occurring *neurotransmitters*. For example, the NMDA receptor is named for N-methyl-D-aspartate, a synthetic compound that has been found to bind and activate the receptor in the same way that the naturally occuring *neurotransmitter* glutamate does.
Phospholipids: A type of lipid, or fat, found in all cells that forms the structural basis of cell membranes.
Polymorphism: The occurrence of two or more different forms of the same genetic trait in a population. For example, in a population of rats that otherwise are genetically identical, one group is sensitive to the effects of alcohol, whereas the other group is much less sensitive. Sensitivity to alcohol’s effects is polymorphic in this rat population.
Regulatory elements: DNA sequences located near a *gene*. These influence protein production from the *gene* by helping to define the rate at which the *gene* is expressed.
Serotonin: One of the primary *neurotransmitters* in the central nervous system that excites target neurons.

## References

[b1-arhw-19-1-60] Mello NK, Mendelson JH (1971). Recent Advances in Studies of Alcoholism: An Interdisciplinary Symposium.

[b2-arhw-19-1-60] Tabakoff B, Petersen RC (1988). Reports from research centers–13: Intramural research program of the National Institute on Alcohol Abuse and Alcoholism. British Journal of Addiction.

